# Human adult bone marrow-derived stem cells decrease severity of lipopolysaccharide-induced acute respiratory distress syndrome in sheep

**DOI:** 10.1186/scrt430

**Published:** 2014-03-26

**Authors:** Mauricio Rojas, Nayra Cárdenes, Ergin Kocyildirim, John R Tedrow, Eder Cáceres, Robert Deans, Anthony Ting, Christian Bermúdez

**Affiliations:** 1 Dorothy P. and Richard P. Simmons Center for Interstitial Lung Diseases, University of Pittsburgh School of Medicine, Pittsburgh, PA 15213, USA; 2 Acute Lung Injury Center of Excellence, University of Pittsburgh School of Medicine, Pittsburgh, PA 15213, USA; 3 Division of Pulmonary, Allergy and Critical Care Medicine, University of Pittsburgh School of Medicine, Pittsburgh, PA 15213, USA; 4 McGowan Institute for Regenerative Medicine, University of Pittsburgh School of Medicine, Pittsburgh, PA 15213, USA; 5 Division of Cardiothoracic Transplantation, University of Pittsburgh School of Medicine, 3459 Fifth Avenue, Pittsburgh, PA 15213, USA; 6 Athersys, Inc., 3201 Carnegie Ave., Cleveland, OH 44115, USA

## Abstract

**Introduction:**

Acute respiratory distress syndrome (ARDS) is the most common cause of respiratory failure among critically ill subjects, sepsis and severe bacterial pneumonia being its most common causes. The only interventions that have proven beneficial are protective ventilation strategies and fluid conservation approaches. New therapies are needed to address this common clinical problem. Others and we have previously shown the beneficial effect of infusion of exogenous adult stem cells in different pre-clinical models of ARDS.

**Methods:**

In the present study endotoxin was infused intravenously into 14 sheep from which 6 received different doses of adult stem cells by intrabronchial delivery to evaluate the effect of stem cell therapy.

**Results:**

After administration of endotoxin, there was a rapid decline in oxygenation to hypoxemic values, indicative of severe-to-moderate ARDS. None of the animals treated with saline solution recovered to normal baseline values during the 6 hours that the animals were followed. In contrast, sheep treated with a dose of 40 million adult stem cells returned their levels of oxygen in their blood to baseline two hours after the cells were infused. Similarly, improvements in carbon dioxide (CO_2_) clearance, pulmonary vascular pressures and inflammation were observed and confirmed by histology and by the decrease in lung edema.

**Conclusions:**

We concluded that instillation of adult non-hematopoietic stem cells can diminish the impact of endotoxin and accelerate recovery of oxygenation, CO_2_ removal and inflammation in the ovine model, making the use of adult stem cells a real alternative for future therapies for ARDS.

## Introduction

Acute respiratory distress syndrome (ARDS) is a common clinical entity and a major cause of morbidity and mortality in the critical care setting [1]. The recent Berlin definition classifies it in three different degrees of severity according to the level of hypoxemia, calculated as: mild*,* 300 to 201 mmHg partial pressure of oxygen (PaO_2_)/fraction of inspired oxygen (FiO_2_); moderate, 200 to 101 mmHg PaO_2_/FIO_2_; and severe, PaO_2_/FIO_2_ ≤ 100 mmHg [2,3].

Although ARDS results from a wide variety of disorders, sepsis is its main cause and the risk factor most associated with high mortality [4-6]. Regardless of the cause, the alveolar epithelium and capillary endothelium are affected, leading to an increase in permeability allowing protein-rich fluid to accumulate in the alveolar space [7-9]. The loss of epithelial integrity disrupts alveolar clearance and production of surfactant [10-12]. In addition to the alveolar damage, there is an influx of circulating inflammatory cells and formation of hyaline membranes usually caused by the mechanical ventilation. If the inflammatory process is severe enough, there will be ensuing disorganized repair resulting in fibrosis [13].

Existing therapy is currently limited to supportive care [14,15]. A novel potential therapy for ARDS is the use of bone marrow-derived mesenchymal stem cells (B-MSC) [16-20]. We have previously demonstrated that the infusion of B-MSC isolated from mice and swine prevented inflammation and aberrant repair of endotoxin-induced lung injury in both species [19,21-23]. These effects, together with the restoration of fluid clearance and the decrease in bacterial growth, have also been shown in an *ex-vivo* perfused human lung model of septic ARDS [16]. There is strong evidence that in models of ARDS, after infusion B-MSC are activated inducing secretion of multiple soluble factors that results in significantly lower levels of inflammatory cytokines in both plasma and bronchoalveolar lavage (BAL) [19,20,24,25]. B-MSC are also able to alter the systemic redox environment characteristic of ARDS to a less oxidizing value [18,26-30] and restore the alveolar epithelium and endothelium integrity and permeability, decreasing airspace neutrophils [30,31]. The transfer of functional mitochondria from B-MSC to the epithelium has been proven instrumental in the repair process of the lung [32-34] and B-MSCs have also been shown to have anti-bacterial effects that are very beneficial in the septic environment [35-38].

We designed a preclinical large animal model of endotoxin-induced ARDS in order to evaluate the safety and efficacy of the use of adult bone marrow-derived stem cells, named MultiStem (Athersys, Cleveland, OH) in the treatment of moderate-to-severe ARDS. In the present study, sheep with lipopolysaccharide (LPS)-induced ARDS received Good Manufacturing Practice (GMP)-MultiStem, which have been used in clinical trials for organs other than the lung [39,40], with no toxicity reported. Our results suggest that MultiStem have the ability to reduce the duration and severity of the injury, without detected secondary toxic effects. This allows us to propose the translation of bone marrow-derived stem cells into clinical studies for the treatment of patients with ARDS.

## Methods

### Animal model

Fourteen adult Dorsett Cross sheep weighing 36.5 to 65 kg were used in the present study. All animals received humane care in compliance with the ‘Principles of Laboratory Animal Care’ formulated by the National Society for Medical Research and the ‘Guide for the Care and Use of Laboratory Animals’ prepared by the Institute of Laboratory Animal Resources and published by the National Institutes of Health (NIH) (NIH no. 86–23). The Institutional Animal Care and Use Committee (IACUC) for Animal Research of the University of Pittsburgh approved all experimental procedures in advance. The use of human stem cells in animals was approved by the Human Stem Cell Research Oversight (hSCRO) Office at the University of Pittsburgh.

A Swan-Ganz catheter was inserted through the jugular vein, after which open chest superior vena cava and main pulmonary artery were cannulated. The sheep received intravenously via the Swan-Ganz catheter 1 and 3.5 μg/kg *E. coli* endotoxin LPS from *E. coli* 055:B5 (Sigma, St. Louis, MO, USA) in normal saline (Baxter, Deerfield, IL, USA) over 30 minutes at 0.7 mL/minute to induce moderate-to-severe ARDS, as defined by the ARDS Definition Task Force [2]. The experimental group (stem cell) received 4, 10 or 40 million MultiStem cells intrabronchially into the lower left lung 30 minutes after the end of LPS infusion. The control group received the same volume intrabronchially of saline (Figure S1 in Additional file 1).

### Adult stem cell isolation and administration

MultiStem were isolated from a human donor bone marrow aspirate. Bone marrow aspirates were acquired with consent and in accordance with 21 CFR Part 1271 Human Cells, Tissues, and Cellular and Tissue Based Products and approved by the Institutional Review Board. Cell isolation was processed according to previously described methods [41]. Briefly, human MultiStem were isolated from a single bone marrow aspirate, obtained with consent from a healthy donor, and cultured in fibronectin-coated plastic tissue culture flasks. Cell cultures were maintained under low oxygen tension in a humidified atmosphere of 5% CO_2_. Cells were cultured in MultiStem culture media: low-glucose (D)MEM (Life Technologies, Grand Island, NY, USA) supplemented with FBS (fetal bovine serum; Atlas, Fort Collins, CO, USA), ITS liquid media supplement (Sigma), MCDB {AU Query: Please spell out what ITS and MCDB stand for followed by (ITS) and (MCBD), respectively.} (Sigma), platelet-derived growth factor (R&D Systems, Minneapolis, MN, USA), epidermal growth factor (R&D Systems), dexamethasone, penicillin/streptomycin (Life Technologies), 2-phospho-L-ascorbic acid and linoleic acid-albumin (Sigma). Cells were passaged every three to four days and harvested using trypsin/ethylenediaminetetraacetic acid (EDTA) (Life Technologies). The cells were positive for CD49c and CD90 and negative for MHC class II and CD45 (all antibodies (Abs) were from BD Biosciences, San Jose, CA, USA). Cells were cryopreserved in MultiStem media and 10% dimethyl sulfoxide (DMSO). Before administration cells were counted with trypan blue exclusion and final concentration adjusted according with the percentage of alive cells. Preparations in which cell viability were lower that 90% were discarded.

### Data acquisition and analysis

In all groups, blood gases and blood sampling were performed in a Radiometer ABL 725 (Radiometer, Westlake, OH, USA) before the endotoxin administration and every 30 minutes for the duration of the study (Figure S1 in Additional file 1). To have a better understanding of the lung function, open chest hemodynamic measurements, aortic pressure (AoP), pulmonary artery pressure (PAP), central venous pressure (CVP), and left atrial pressure (LAP) were monitored with catheter-tip manometers (Additional file 1 and Figure S2 in Additional file 1). Cardiac output (CO) was monitored continuously. All hemodynamic parameters were recorded electronically in a secure access hard drive for further analysis.

### Histopathologic evaluation

**Figure 1:**
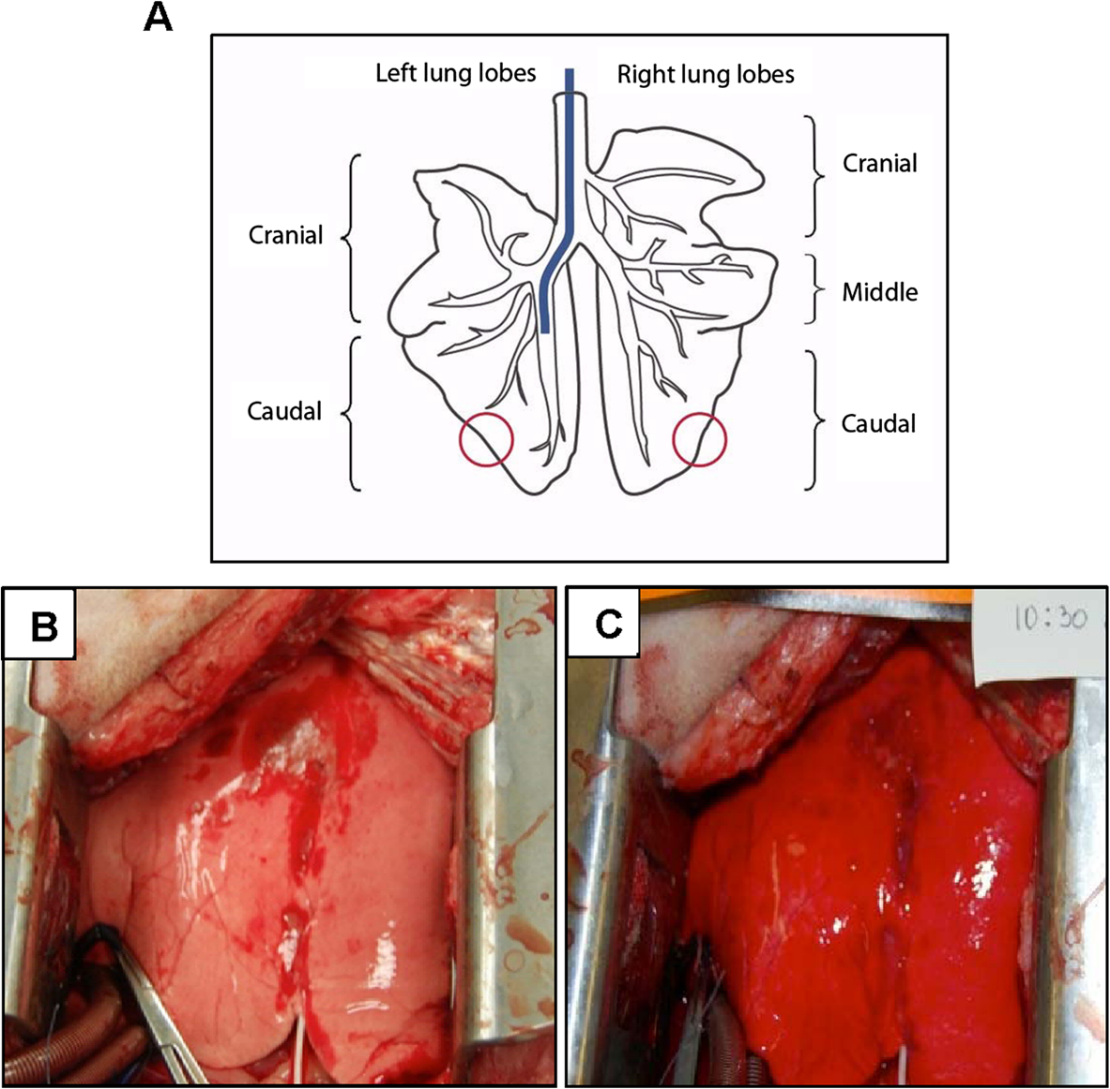
**Sheep lung diagram and pictures. (A)** The blue line represents the path of the bronchoscope to deliver the adult stem cells or saline. The red circumferences show the areas where the lung tissues were sampled. Lung congestion and edema are visually apparent as a consequence of endotoxin infusion. Pictures of the left lung of a sheep before **(B)** and one hour after the end of the infusion of endotoxin **(C)**.

Lung biopsies of 1 to 2 cm^3^ were performed at the lower lobes (left and right) before and after endotoxin and at the end of the experiment (Figure 1A). A piece of each biopsy was fixed with 10% non-buffered formalin for 24 hours, paraffinized and sectioned for subsequent staining with hematoxylin and eosin for histological assessment using light microscopy (Nikon Eclipse 55i, Melville, NY, USA). The rest of the tissue was used for wet-to-dry analysis.

### Wet to dry ratio

Pieces of the biopsies from the lower lobes of the lungs from each of the three time points described were divided into five 2 to 3 mm^2^ fragments each for use as technical replicates and flash frozen separately for later weighing. The tissues were dried overnight in a Savant DNA120 SpeedVac Concentrator (ThermoFisher, Pittsburgh, PA, USA) and reweighed, then the wet-to-dry ratio (W/D) was determined as the percent change after injury, $\frac{\left(\frac{W}{D}\right)f}{\left(\frac{W}{D}\right)t}100-100$ (where *W* is the wet weight, *D* is the dry weight, *f* is the final biopsy at the end of the study and *i* the biopsy one hour after endotoxin).

### Statistical analysis

Comparison between the control group and the groups treated with MultiStem, as well as the comparison between baseline and the following time points within a group were tested with independent student *t*-tests. The differences in the results were considered to be statistically significant when the *P*-value was less than 0.05.

## Results and discussion

We were able to demonstrate morphological and physiological changes consistent with moderate-to-severe ARDS as a consequence of systemic administration of a single dose of endotoxin. After endotoxin infusion, lung congestion and edema were visually apparent, where accumulation of blood and fluid in the lungs is clearly recognized (Figure 1B-C). The observed changes reached a peak one hour after the end of the infusion of the endotoxin.

In the initial reports by our group, we delivered intravenously B-MSCs to mice and swine with LPS-induced lung injury demonstrating protection with non- or minimal engraftment [19,25,30]. In the present work, we decided to deliver a similar cell type intratracheally. The rationale for the change in the route of delivery was based on the possible clinical use of the cells by reducing possible secondary effects. During ARDS, in addition to the deteriorated pulmonary function, there is accumulation of circulatory cells by an increase in the adherence to the endothelium. Furthermore, the large size of the mesenchymal stem cells (close to 100 μm in diameter) that circulate in the 10 μm microvessels of the lung increases the risk of pulmonary microemboli [42]. In addition, we decided to deliver the cells into only one lung leaving the other lung intact to reduce possible major complications. However, it is important that several studies have demonstrated that during lung injury intravenous and intratracheal administration of mesenchymal stem cells can prompt lung protection [16,43].

### Blood gas and hemodynamic data

**Figure 2:**
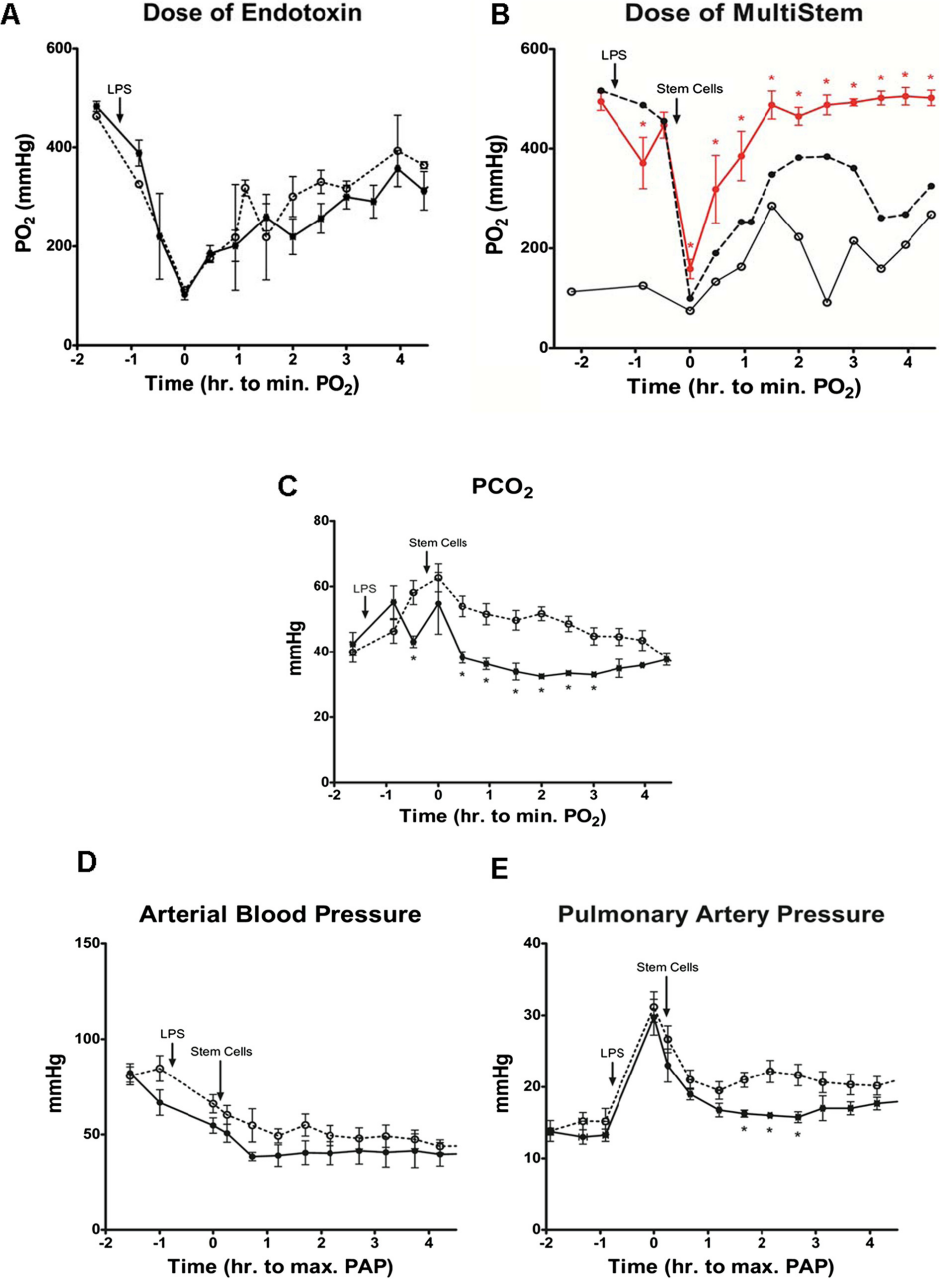
**Treatment with stem cells facilitate the return to arterial basal levels of blood gases and hemodynamics. (A)** The most appropriate dose of endotoxin for the control group (no treatment) was first determined; 1 (solid line) and 3.5 μg/kg (dotted line). **(B)** Similarly, the dose of MultiStem for the experimental group was determined according to their ability to maintain the PO_2_ levels after instillation. Doses of 4 (solid line), 10 (dotted line) and 40 million (red line) MultiStem were delivered intrabronchially a half hour after the end of LPS injection. The time of highest pulmonary injury (lowest arterial PO_2_) is expressed as time zero. **(C)** Measurement of the PCO_2_ levels similarly demonstrated a protective effect in the experimental group (solid line) compared to the control (dotted line). Arterial **(D)** and pulmonary artery **(E)** pressures stabilize earlier in animals treated with stem cells. The control group (no treatment) is represented with a dotted line. Forty million stem cells were instilled intrabronchially in the experimental group (solid black) 30 minutes after the end of LPS injection. The time of highest PAP is expressed as time zero. LPS, lipopolysaccharide; PAP, pulmonary artery pressure; PCO_2_, partial pressure of CO_2_.

Blood oxygenation and CO_2_ clearance are the primary functions of the lung. One of the main characteristics that defines ARDS is a deficit in these functions, where oxygenation capacity is impaired and the hypoxemia level falls below a PaO_2_/FiO_2_ ratio of 300 mmHg [2]. In this animal model we reproduced the pathophysiology of ARDS observed in humans by administering bacterial endotoxin systemically in sheep. We first evaluated the effect of endotoxin on changes in lung function; as we show in Figure 2A, endotoxin was administered intravenously and blood gas analysis demonstrated an induction of moderate-to-severe ARDS by the PaO_2_/FiO_2_ levels. We evaluated the effect of two different doses of endotoxin, 1 and 3.5 μg/ml intravenously. With both concentrations we observed a rapid decrease in the PaO_2_, reaching its minimum one hour after the end of endotoxin infusion followed by steady recovery. PaO_2_/FiO_2_ values in both groups dropped 77 ± 10% (standard error, SE) from baseline level at its minimum (102 ± 10 mmHg). We decided to use the higher dose because of the reproducibility between experiments and because after five hours the recovery was only partial.

Similarly, multiple doses of MultiStem were used before selecting the most appropriate. As shown in Figure 2B, doses of 2 and 10 million cells failed to induce significant protection. There was only a marginal effect, when a dose of 10 million was used. In contrast, 40 million cells clearly were able to accelerate the recovery. This group displayed PaO_2_/FiO_2_ values significantly higher than the control group throughout the experiment (*P* <0.02) and reached baseline levels less than three hours after the endotoxin infusion, whereas the control group only recovered to 80% of baseline level by the end of the study while the stem cell group’s lowest value was significantly higher (68 ± 12% SE lower than baseline, 158 ± 19 mmHg; *P* <0.024). From this point, the data presented in the experimental group will be limited to the dose of 40 million.

Immediately after the drop in PaO_2_/FiO_2_, the partial pressure of CO_2_ (PCO_2_) levels increased comparably in both groups. Nevertheless, half an hour after maximum lung injury (minimum PaO_2_/FiO_2_), the experimental group reached the PCO_2_ baseline level, while the control group stayed higher than the baseline throughout the study (*P* < 0.005) (Figure 2C). The stem cell treated animals were followed for more than two hours after returning to baseline, suggesting that there was no need for longer monitoring.

After endotoxin infusion, most of the hemodynamic values showed important changes, with the exception of the heart rate which maintained baseline values in both groups (data not shown). Mean arterial pressure decreased significantly (*P* <0.025) and remained below baseline values throughout the study duration in both groups (Figure 2D). CVP was increased in the control group after endotoxin without significant differences; however, the experimental group treated with stem cells maintained baseline values throughout the study (data not shown). In the control group, PAP stayed significantly higher than baseline (51 ± 2%) after the endotoxin infusion (*P* <0.02) throughout the study, whereas two hours after endotoxin, the experimental group had recovered to 24 ± 2% higher than baseline values (Figure 2E). CO showed a strong decreasing tendency after endotoxin infusion, without reaching the level of significance (data not shown).

In addition, a well-known complication in patients who required assisted ventilation is ventilator-induced lung injury. In our protocol we used protective ventilation, but we cannot discount that some of the injury we observed was induced during ventilation. However, it has been documented that mesenchymal stem cells have the ability to protect the lungs in rats with ventilator induced lung injury [43]. This may suggest a double protective effect of the MultiStem.

### Inflammatory data

**Figure 3:**
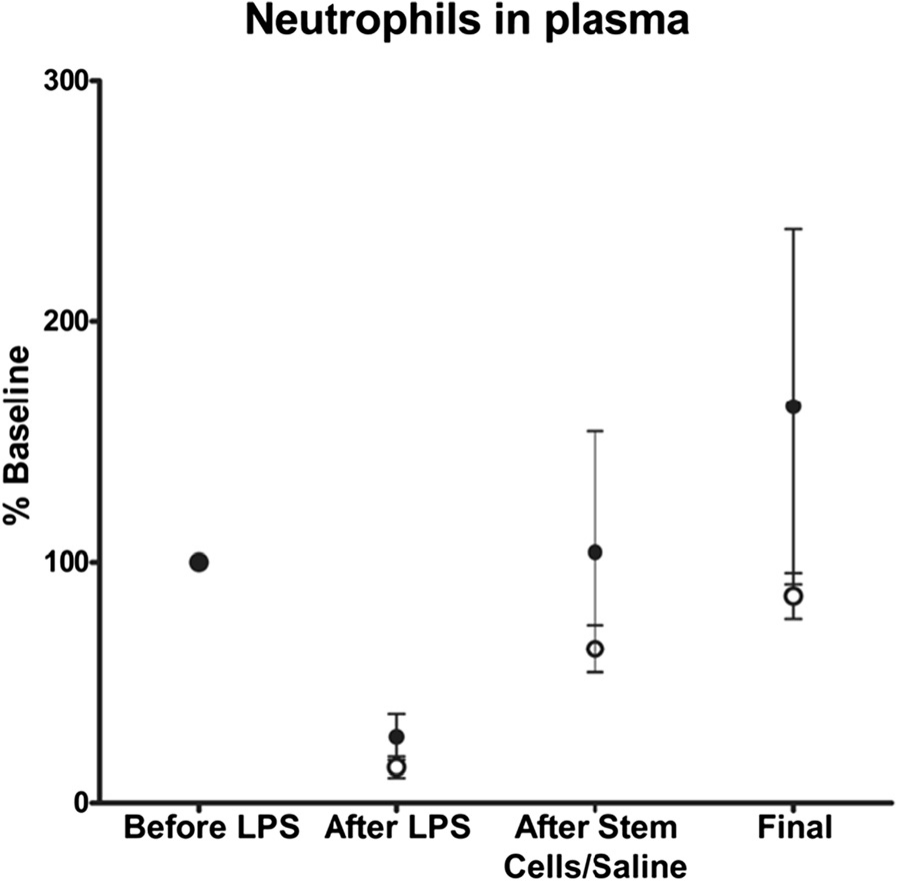
**Neutrophils in plasma tend to increase more in animals treated with stem cells after endotoxin injury.** The control group (no treatment) is represented with open circles and the experimental group (receiving stem cells) is shown in black circles. Values are shown as percent baseline.

**Figure 4:**
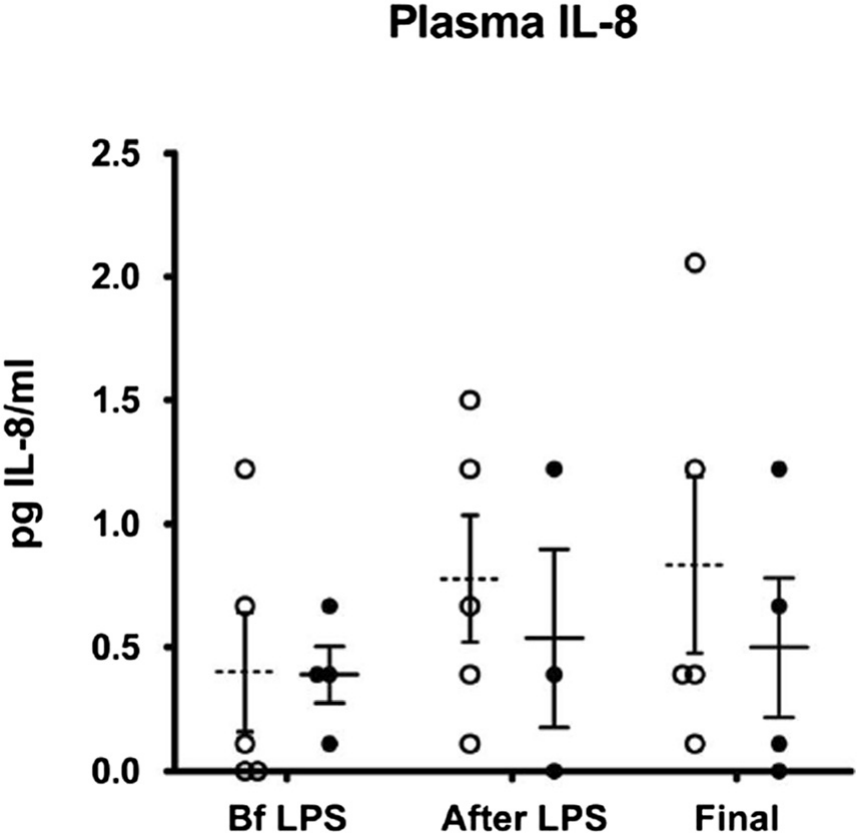
**Data suggest a decrease in IL-8 at the end of the experiment in animals that received adult stem cells one hour after LPS.** Detection of sheep IL-8 in serum was by ELISA. Values from animals that received saline as control are depicted in open circles, and values from animals that received stem cells are shown in black circles. LPS, lipopolysaccharide.

ARDS is characterized by the sequestration of neutrophils in the lung, which results in a low neutrophil count in the circulation. As expected, significantly lower than baseline values were recorded in plasma in both groups after the endotoxin infusion (*P* <0.0003). The stem cell group recovered to baseline levels of neutrophil counts after the infusion of cells while the control group did not recover until the end of the study (Figure 3). This was consistent with lower plasma levels of the pro-inflammatory cytokine IL-8, in which plasma levels after injury were higher in the control group compared to levels in the experimental group (Figure 4). IL-6 cytokine was also measured in plasma, but the values did not reach the detection range (data not shown).

### Lung edema

One of the hallmarks of ARDS is the accumulation of alveolar edema due to impaired alveolar fluid clearance and increased microvascular permeability of the lung endothelium, and it is often used as a prognostic tool for morbidity and mortality [44,45]. It has been shown by others and us that lung edema is significantly abrogated in animals treated with MSC or leukocyte-depleted bone marrow [19,20]. Here we also show an improvement in lung edema upon administration of adult stem cells.

**Figure 5:**
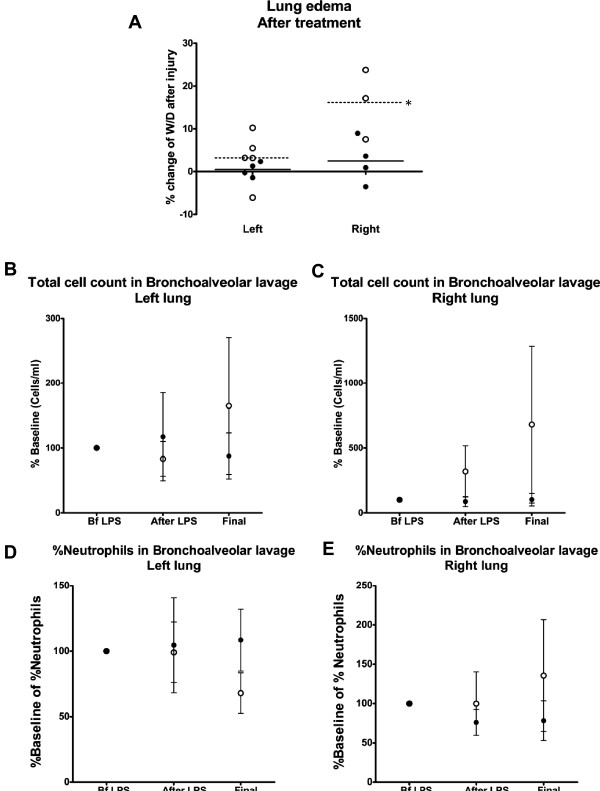
**Pulmonary edema is prevented in animals instilled with adult stem cells after endotoxin injury. (A)** Tissue samples were collected right before and after endotoxin infusion and at the end of the experiment from both lungs. Water content was measured by calculating the weight before and after the tissue was dehydrated, and the ratio was then calculated. Each sample consisted of five replicates. The control group is represented by open circles and the stem cell group by black circles. Total cell number and neutrophil concentration in bronchoalveolar lavage (BAL) tend to be higher in control animals than in those treated with stem cells. Left **(B,D)** and right **(C,E)** lungs. The total cell count in BAL **(B,C)** and the percent of neutrophils **(D,E)** are shown for time points before (Bf) and after endotoxin infusion and at the end of the study. The control group (no treatment) is represented with open circles and the experimental group (receiving stem cells) is shown in black circles.

Lung edema was analyzed by W/D of lower lobe lung biopsies. The control group showed an increase of relative water content after injury in both lungs, reaching significant differences in the right lung (*P* <0.001), while treatment with stem cells prevented the increase in edema in either lung (Figure 5A).

### Bronchoalveolar lavage

#### *Left lung data*

Figure 5 (B,D) shows the series of BAL findings in the left lung (site of cell instillation). Total cell count was observed to be low in both groups after the endotoxin infusion and showed a slight tendency to decrease throughout the study duration. The percentage of neutrophil count had a stable constant trend throughout the study and was detected at lower than baseline values at the end of the study in the control group. The percentage of lymphocytes and monocytes was stable throughout the study (data not shown). None of the changes in the left lung BAL findings reached the level of statistical significance.

#### *Right lung data*

Figure 5 (C,E) shows the series of BAL findings in the right lung. Total cell count and neutrophil percentage remained stable in both groups. Lymphocytes were stable and monocytes showed a slight tendency to increase after endotoxin and then came back to baseline in the control group. None of the BAL findings in the right lung reached the level of significance.

### Histopathologic data

#### *Left lung*

**Figure 6:**
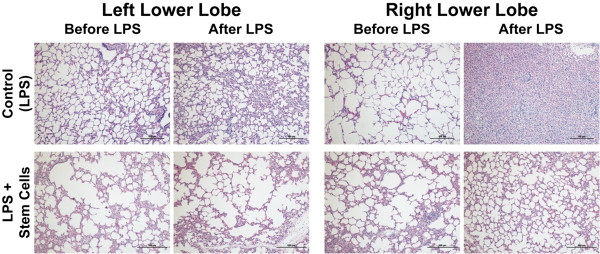
**Histological sections of left and right lungs of sheep treated with bacterial endotoxin show less inflammation and congestion in the lungs treated with stem cells.** Lung biopsies were fixed in 10% formalin and stained with hematoxylin and eosin (H&E; magnification 10x). Sections are shown for left and right lungs before endotoxin infusion and at the end of the study of representative animals of each group (control (LPS) and LPS + stem cells). LPS, lipopolysaccharide.

Histopathologic evaluation revealed increased inflammatory cellular infiltration in the control group after the endotoxin infusion. Interstitial acute inflammation with edema, and neutrophil infiltration was evident, while reduced inflammation was recorded before the end of the study in the group treated with stem cells (Figure 6).

#### *Right lung*

Histopathologic changes were noted to be severe in the right lung in comparison to the left. As observed in the left lung, reduced inflammation was recorded in the experimental group before the termination of the study (Figure 6).

An interesting observation is the differences in the severity of the injury in the right lung between animals treated or not with MultiStem. The fact that the animals are reposing on their right, under normal conditions during this short period of time fluid accumulation is not detected. However, in the case of LPS-induced ARDS where there are hemodynamic and respiratory failures, fluid cannot be removed which results in severe histological changes and an increase in water counted.

### Safety of the intrabronchial delivery of MultiStem in the sheep ARDS model

**Figure 7:**
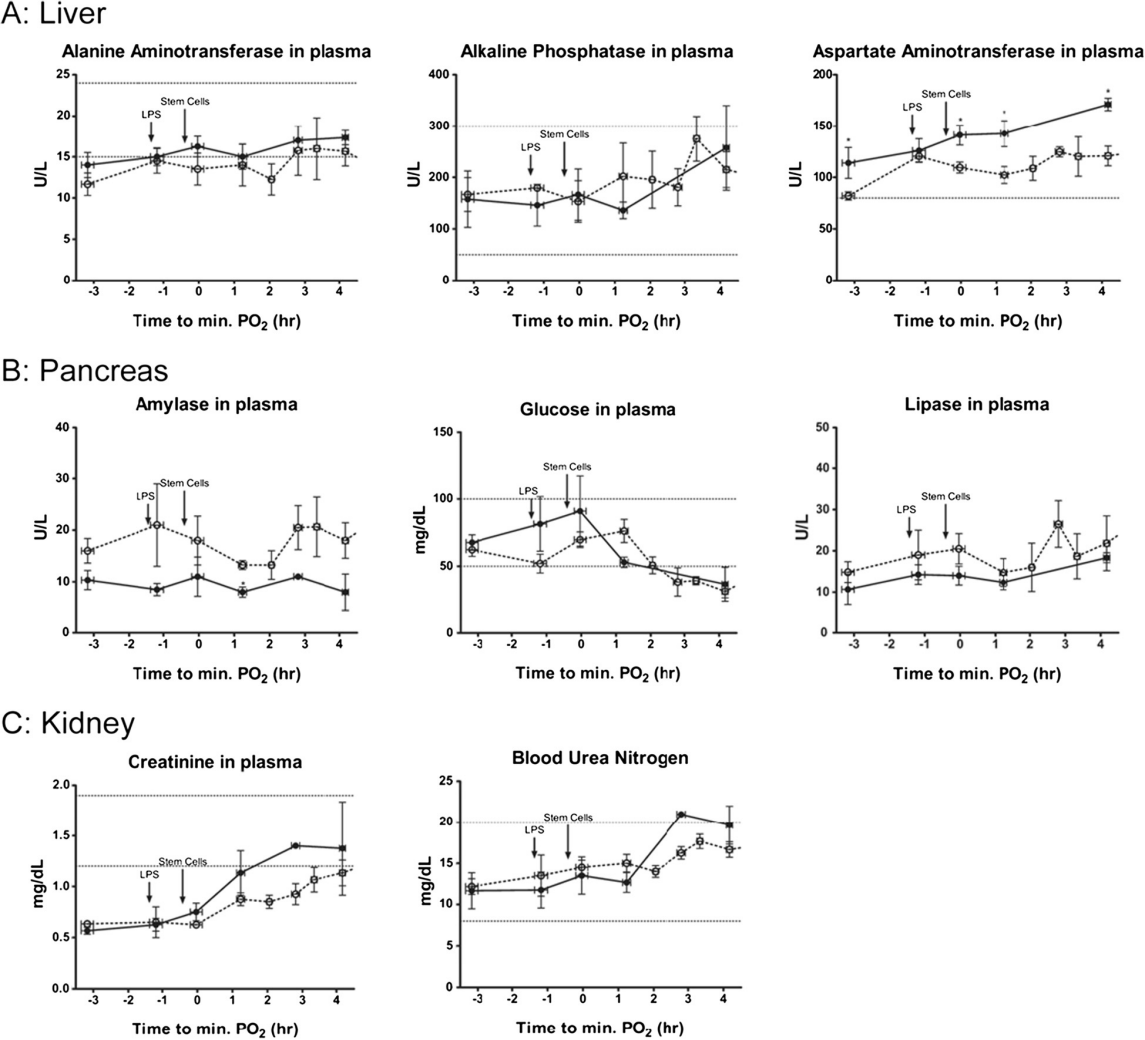
**Liver, pancreas and kidney data show no organ toxicity after the intrabronchial infusion of adult stem cells during endotoxin-induced ARDS.** To measure liver function **(A)**, protein levels of alanine aminotransferase, alkaline phosphatase and aspartate aminotransferase were measured in plasma. Amylase, glucose and lipase plasma levels were quantitated to evaluate pancreatic function **(B)** and creatinine and blood urea nitrogen for kidney function **(C)**. All the values are within normal ranges. The control group (no treatment) is represented by dotted lines and the experimental group (receiving stem cells) by solid black lines. ARDS, acute respiratory distress syndrome.

As part of this pre-clinical study, several markers of organ function were measured, including in the liver (Figure 7A), pancreas (Figure 7B) and kidney (Figure 7C). Plasma levels of alanine aminotransferase, alkaline phosphatase and aspartate aminotransferase (ASAT) were measured for evaluation of liver function. Plasma levels of amylase, glucose and lipase were quantitated to evaluate pancreatic function, and creatinine and blood urea nitrogen for kidney function. Recorded values fell within the normal ranges expected for sheep. We observed statistical differences in ASAT levels. However, because they were within normal values, these differences do not have biological significance. These results indicate that there was no organ-associated toxicity related to the administration of the MultiStem.

## Conclusions

In order to assess the effectiveness and safety of adult stem cells for the treatment of ARDS, we developed a short-term sheep preclinical model by systemic infusion of endotoxin that resulted in a moderate-to-severe ARDS. The considerable experimental literature demonstrating the protective effects of adult stem cells (for example, B-MSC) in models of ARDS [19,23,46-51], together with the clinical safety experience of MultiStem in previous trials, indicates that these cells will be well-tolerated in the critically ill ARDS population.

The mechanisms by which adult stem cells participate in the repair of the lung following injury have been attributed to their different qualities [16,25,52,53]. The ability of the cells to secrete paracrine factors, such as growth factors and anti-inflammatory cytokines, and to control oxidative damage, protect the endothelium and epithelium, transfer functional mitochondria and secrete antimicrobial peptides has been demonstrated to explain some of the therapeutic effects in the treatment of lung injury in animal *in vivo* and *ex vivo* models. Previous studies by our group and others have demonstrated the beneficial effects of the administration of exogenous B-MSC in endotoxemic mice. These studies repeatedly showed a decrease in systemic and local lung inflammation and lung injury [19,20,23,24], demonstrating the anti-inflammatory effect of these cells. Additionally, the immunomodulatory effects and low immunogenicity described for adult stem cells make them promising candidates for therapy. In this study, we show through histologic staining, plasma neutrophil count and plasma pro-inflammatory IL-8 cytokine levels that there was a marked decrease in inflammation affected by the use of adult stem cells. Is a single dose or multiple doses of MultiStem the most appropriate to treat ARDS? We believe that will, in the future, be a particular decision for each individual patient.

One of the most profound effects of endotoxemia is a decrease in the levels of oxygen in circulation. In our acute preparation, local instillation of stem cells was able to reduce the severity and decrease the duration of the injury. Although the arterial blood pressure remained low throughout the study in both groups, the infusion of stem cells in ARDS-induced animals attenuated the endotoxemia, as PAP was stabilized to within 20% of baseline and the control only recovered to 50%.

An important observation was the dramatic distal effect that cells delivered into the left lung had on the right lung. We have two possible explanations. First, because animals are on right lateral recumbency, this favors fluid accumulation. Under normal conditions water accumulation cannot be detected. However, because organ failure during the LPS-induced ARDS and by lost in endothelial barrier there more fluids are produced and this associated with a decrease in normal hemodynamic function results in a ‘perfect storm’ with a massive accumulation of fluids and inflammatory cells. Second, distal effect can be explained by the secretion of soluble factors by the MultiStem, this paracrine effect has been well documented. Cells injected intravenously accumulate in the lung and have distal effects on the heart and eye [54,55]. It is possible that the observed results are the combination of these two mechanisms.

Furthermore, we monitored organ function of the liver, pancreas and kidney to evaluate the possible toxic effects of the cells. Protein plasma levels fell within normal values for sheep, suggesting no organ-associated toxicity related to the intrabronchial administration of MultiStem during ARDS. Therefore, the use of these cells is considered to be safe in the ARDS model.

Overall, the local administration of adult stem cells to a systemic endotoxemic insult in sheep appears, in the short term, to improve lung function, inflammatory response and hemodynamic changes produced by the bacterial toxin without affecting other organs. Thus, we believe that adult stem cells are a promising candidate for a novel therapy that will help lower the mortality rate in ARDS patients, reducing the associated complications and subsequent multi-organ failure characteristic of this syndrome.

## Abbreviations

ARDS: acute respiratory distress syndrome; BAL: bronchoalveolar lavage; B-MSC: bone marrow-derived mesenchymal stem cells; CO: cardiac output; CVP: central venous pressure; FiO2: fraction of inspired oxygen; IL: interleukin; LPS: lipopolysaccharide; PaO2: partial pressure of oxygen; PAP: pulmonary artery pressure; W/D: wet to dry ratio.

## Competing interests

MR, NC, EK, JRT, EC and CB declare that they have no competing interests. RD and AT declare a competing interest; they are employed by Athersys, which provided the cells.

## Authors’ contributions

MR and CB were involved in the conception, design, acquisition of data, analysis, interpretation of data and drafting of the manuscript, and agree to be accountable for all aspects of the work in ensuring that questions related to the accuracy or integrity of any part of the work are appropriately investigated and resolved. NC was involved in the acquisition of data, analysis, interpretation of data and drafting of the manuscript, and agrees to be accountable for all aspects of the work in ensuring that questions related to the accuracy or integrity of any part of the work are appropriately investigated and resolved. EK, JRT and EC have made substantial contributions in the acquisition and interpretation of the data and agree to be accountable for all aspects of the work. EK, JRT, EC, RD and AT have been involved in the conception and design of the study and in drafting of the manuscript and revising it critically for important intellectual content. All authors have read and approved the final manuscript.

## Supplementary Material

Additional file 1: Figure S1 Experimental model of lung injury in sheep to assess the use of bone marrow derived stem cells. The control and the stem cell groups consisted of six and four sheep, respectively. The ARDS was induced by intravenous infusion of 3.5 μg/kg bacterial endotoxin (LPS). Three bronchoalveolar lavages (BALs) and lung biopsies were performed before endotoxin infusion (baseline), one hour after and at the end of the study. Peripheral blood samples were collected before the infusion and every hour after (time points illustrated with red dots). The experimental group received a dose of 40 million bone marrow-derived stem cells intrabronchially one hour after the start of endotoxin infusion, the control group received a corresponding volume of saline. **Figure S2.** Diagram of the cannulation in the open chest preparation. The pulmonary artery, left atrium and pulmonary artery are indicated. Click here for file
