# Supplementary material for: Human adult bone marrow-derived stem cells decrease severity of lipopolysaccharide-induced acute respiratory distress syndrome in sheep
Source: Stem Cell Res Ther. 2014 Mar 26;5(2):42. doi: 10.1186/scrt430 (PMC4055116; doi:10.1186/scrt430)
Supplement: Additional file 1: Figure S1 — Experimental model of lung injury in sheep to assess the use of bone marrow derived stem cells. The control and the stem cell groups consisted of six and four sheep, respectively. The ARDS was induced by intravenous infusion of 3.5 μg/kg bacterial endotoxin (LPS). Three bronchoalveolar lavages (BALs) and lung biopsies were performed before endotoxin infusion (baseline), one hour after and at the end of the study. Peripheral blood samples were collected before the infusion and every hour after (time points illustrated with red dots). The experimental group received a dose of 40 million bone marrow-derived stem cells intrabronchially one hour after the start of endotoxin infusion, the control group received a corresponding volume of saline. Figure S2. Diagram of the cannulation in the open chest preparation. The pulmonary artery, left atrium and pulmonary artery are indicated. [file scrt430-S1.pdf]

Human Adult Bone Marrow-Derived Stem Cells Decrease Severity of LPS-Induced Acute Respiratory Distress Syndrome in Sheep

Mauricio Rojas, Nayra Cárdenes, Ergin Kocyildirim,John R. Tedrow, Militza R. Romagnoli, Eder Cáceres, Robert Dean, Anthony Ting and Christian Bermúdez

SUPPLEMENTAL DATA

Supplemental Methods

Operative Procedures

Each sheep was pre-medicated with intramuscular ketamine (25-30 mg/Kg; Henry Schein, Dublin, OH). General anesthesia was maintained with isoflurane (2.5-3%). They were initially ventilated with a mixture of room air and oxygen (fraction of inspired oxygen of 60%) at a frequency of 12 to 15 breaths/min and a tidal volume starting at 10 mL/kg/min. The settings were adjusted by maintaining arterial partial CO_2 pressure of 35-40 mmHg. Upon administration of endotoxin, the sheep were ventilated with a fraction of inspired oxygen (FiO_2) of 100%.

The animals were placed in left lateral recumbency for cannulation of the right carotid artery for recording aortic pressure (AoP) and blood sampling. A Swan-Ganz catheter was inserted through the jugular vein for recording central venous pressure (CVP), pulmonary artery pressure (PAP) and cardiac output (CO). Saline solution (Baxter; Deerfield, IL) was used for basic intravenous volume substitution at a rate of 1 mL/Kg/min. Neither catecholamines nor other hormonal or pressor substances were administered. Rectal temperatures and standard peripheral electrocardiographic results were monitored continuously.

Animals were switched to right lateral recumbency to perform left thoracotomy in the 4th intercostal space. Thymus was partially removed, pericardiotomy performed and the great vessels were dissected and isolated. 4/0 Ti-CronTM purse strings (Covidien; Dominican Republic) were placed on the superior vena cava (SVC) and main

pulmonary artery (MPA). Heparin (App Pharmaceuticals; Schaumburgh, IL) was administered to keep the activated clotting time above 200 seconds. A 16-gauge radiopaque polyethylene catheter (Terumo; Somerset, NJ) was introduced into the left atrium (LA) to collect blood samples and continuously measure the LA pressures (**Figure Sup 2**).

Bronchoscopy

Bronchoscopy was performed using a fiberoptic endoscope (Olympus GIFXP-160) inserted through a swivel connected to the endotracheal tube in intubated animals while breathing 100% oxygen. The bronchoscopy was wedged in the left and right lower lobes and 30 mL aliquots of 0.9% NaCl at room temperature were instilled and recovered by gentle hand suction. Total cell counts were performed manually in a hemocytometer, and differential cell counts were read in an IDEXX LaserCyte DXBP 013961 Analyzer (IDEXX, U.S.A.).

IL-8 and IL-6 ELISA

Sandwich enzyme immunoassays were used for IL-8 and IL-6 quantification in plasma according to manufacturer's instructions (Genorise, Philadelphia, U.S.A.).

Figure Sup 1

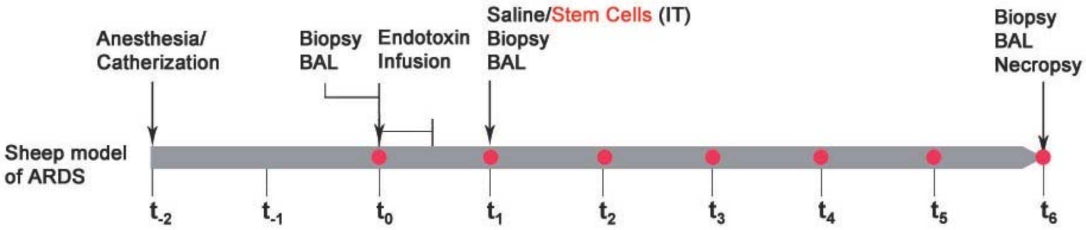

Figure Sup 1. Experimental model of lung injury in sheep to assess the use of bone marrow derived stem cells. The control and the stem cell groups consisted of six and four sheep respectively. The ARDS was induced by intravenous infusion of 3.5μg/Kg bacterial endotoxin (LPS). Three bronchoalveolar lavages (BALs) and lung biopsies were performed before endotoxin infusion (baseline), 1 hr after and at the end of the study. Peripheral blood samples were collected before the infusion and every hour after (time points illustrated with red dots). The experimental group received a dose of 40 million bone marrow-derived stem cells intrabronchially one hour after the start of endotoxin infusion, the control group received a corresponding volume of saline.

Figure Sup 2

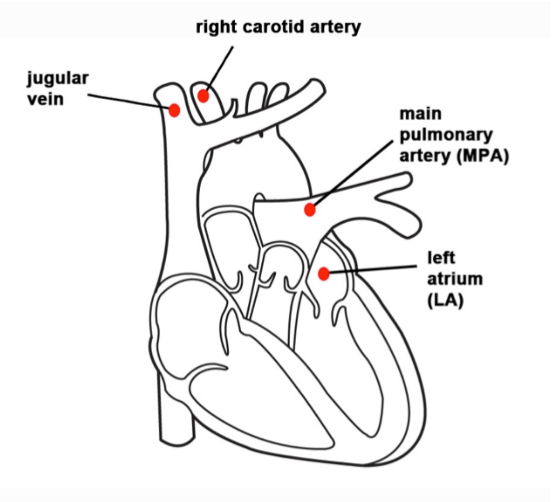

Figure Sup 2. Diagram of the cannulation in the open chest preparation. The Pulmonary Artery, Left Atrium and Pulmonary Artery are indicated.
